# COVID-19 Induced Hepatitis B Virus Reactivation: A Novel Case From the United Arab Emirates

**DOI:** 10.7759/cureus.8645

**Published:** 2020-06-15

**Authors:** Wafa A Aldhaleei, Abdulaziz Alnuaimi, Akshaya S Bhagavathula

**Affiliations:** 1 Gastroenterology, Sheikh Shakhbout Medical City, Abu Dhabi, ARE; 2 Gastroenterology, Abu Dhabi Health Services Company (SEHA), Abu Dhabi, ARE; 3 Public Health, Institute of Public Health, College of Medicine and Health Sciences, United Arab Emirates University, Al Ain, ARE

**Keywords:** novel coronavirus, viral hepatitis b, covid 19, acute on chronic liver disease, hepatitis virus, liver function, adult gastroenterology, hepatology, viral infection, uae

## Abstract

The novel coronavirus disease 2019 (COVID-19) clinically manifests as respiratory and gastrointestinal presentations, most commonly vomiting, diarrhea, and abdominal pain. Although the impaired liver function is prevalent in COVID-19, it is poorly understood. We report the first case of hepatitis B virus (HBV) reactivation caused by COVID-19 in a young adult with altered mental status and severe transaminitis. The patient was asymptomatic, hypothermic, his skin was jaundiced with the icteric sclera, with very high levels of aspartate aminotransferase (AST; 4,933 U/L), alanine aminotransferase (ALT; 4,758 U/L), and total bilirubin (183.9 mmol/L) levels. It is warranted that patients with abnormal liver functions tend to have an increased risk of COVID-19. Thus, increased attention should be paid to the care of patients with abnormal liver functions, and testing for severe acute respiratory syndrome coronavirus 2 (SARS-CoV-2) RNA is warranted in the COVID era.

## Introduction

The novel coronavirus disease 2019 (COVID-19) pandemic is rapidly spreading at an unprecedented rate. Several studies have described the respiratory and gastrointestinal presentations of COVID-19 [1-5]. Fever, cough, dyspnea, and gastrointestinal symptoms such as vomiting, diarrhea, abdominal pain, and elevated liver enzyme and/or bilirubin levels have been reported [2, 6-11]. According to the American College of Gastroenterology, 20-30% of patients with COVID-19 have abnormal liver enzyme levels [12].

Liver impairment resulting in abnormal levels of alanine aminotransferase (ALT) and aspartate aminotransferase (AST) is reported in 14-53% of patients with COVID-19 [13, 14]. Huang et al. reported elevated AST levels in 62% of patients treated in the intensive care unit (ICU) compared with levels seen in non-ICU patients (25%) [5]. Furthermore, AST levels were significantly lower in patients with COVID-19 during the subclinical phase [14].

Severe acute respiratory syndrome-coronavirus-2 (SARS-CoV-2) has a strong affinity for the angiotensin-converting enzyme 2 (ACE2) receptor, which is expressed on multiple cell types, including hepatocytes and cholangiocytes [15]. In this regard, the upregulation of ACE2 receptors in the liver may contribute to the abnormal activities of liver enzyme seen in patients with COVID-19. We report the first case of COVID-19-induced reactivation of hepatitis B virus (HBV).

## Case presentation

A previously healthy 36-year-old man presented to the emergency department with an altered mental status. Two days prior to his hospitalization, the patient experienced intermittent vomiting, with two to three episodes that were reported to a local physician. As the patient progressed to unconsciousness, he was immediately transported to the emergency department. There was no history of fever, cough, shortness of breath, or sensitivity to light. Furthermore, there was no recent or remote history of travel, medication or herbal use, previous surgery, or sick contact with individuals with COVID-19. He did not have a history of consuming alcohol, drug abuse, or unprotected sexual intercourse. On admission, the patient was isolated and tested for COVID-19.

At the initial presentation, physical examination revealed hypothermia of 35.5°C, a pulse of 88/min, blood pressure of 136/78 mmHg, and respiratory rate of 20/min with a room air oxygen saturation of 98%. His skin was jaundiced with the icteric sclera. The liver edge was not palpable and did not reveal hepatomegaly. Chest and cardiovascular examinations were normal. His Glasgow Coma Scale (GCS) score was 7/15.

Pertinent laboratory findings are presented in Table 1. Briefly, the findings were: AST 4,933 U/L (reference: <50 U/L), ALT 4,758 U/L (reference: <40 U/L), total bilirubin 183.9 mmol/L (reference: <21 mmol/L) with direct bilirubin 145 mmol/L (reference: <5 mmol/L), alkaline phosphatase (ALP) 212 U/L (reference: 40-129 U/L), and albumin 33 g/L (reference: 35-52 g/L) (see Table 1). In addition, his international normalized ratio (INR) was >10 (reference: 0.82-1.20). Except for ammonia 74 mmol/L (reference: 16-60 mmol/L), results for all other laboratory assessments such as complete blood count, total protein, and serum chemistry were unremarkable. Computerized tomography (CT) scan of the brain, chest X-ray, and Doppler ultrasound of the liver were normal.

**Table 1:** Liver function test results during hospitalization AST - aspartate aminotransferase; ALT - alanine aminotransferase; ALP - alkaline phosphatase; GGT - gamma-glutamyl transferase; INR - international normalized ratio

Laboratory investigations	Day 1	Day 3	Day 7	Day 16
Total bilirubin (reference: <=21.0 micromol/L)	183.9 ­	232 ­	270 ­	277 ­
Direct bilirubin (reference: <=5.0 micromol/L)	145.0 ­	194 ­	249 ­	243 ­
AST (reference: <=41 IU/L)	4,933 ­	1,204 ­	332 ­	289 ­
ALT (reference: <=40 IU/L)	4,758 ­	2,148 ­	548 ­	212 ­
ALP (reference: 40 -129 IU/L)	212 ­	170 ­	133 ­	135 ­
GGT (reference: 10-66 IU/L)	129 ­	-	-	-
Albumin (reference: 35-52 g/L)	33 ¯	27 ¯	34 ¯	26 ¯
INR (reference: 0.82- 1.20)	>10 ­	3.4 ­	1.3 ­	1.1 (N)

The differential diagnoses for active viral hepatitis (A, C, and E), HIV, autoimmune hepatitis, Wilson’s disease, cytomegalovirus, and Epstein-Barr virus antibodies were negative. The patient tested negative for HIV, hepatitis A, hepatitis C, hepatitis E, cytomegalovirus, and Epstein-Barr virus antibodies. Additional liver workup revealed hepatitis B (HB) surface Ag-positivity, HB core Ab-positivity (IgM), HB envelop (HBe) Ag-negativity, and HBe Ab positivity, suggestive of reactivation of an HBV infection. His hepatitis B DNA viral load was 2,490 IU/mL (reference: <1000 IU/mL), confirming the diagnosis of an acute HBV infection. His COVID-19 polymerase chain reaction (PCR) test results were also positive.

Due to the altered mental status and elevated enzymatic activities in the liver, admission into the ICU was initiated. The patient was started on lactulose via a nasogastric tube (NGT) and was administered cryoprecipitate on day 1. On day 2, entecavir (1 mg daily), vitamin K (10 mg daily), and intravenous thiamin (100 mg daily) administration were initiated. On day 3, his GCS improved to 11/15, with a gradual improvement in liver function parameters. Subsequently, he was shifted to the COVID-19 ward. On day 7, the patient regained complete consciousness (GCS: 15/15); his liver function parameters showed improvements with respect to transaminase and bilirubin levels (Table 1). Multiple nasopharyngeal samples (3/4) for the SARS-CoV-2 PCR tested positive, but his chest X-ray remained normal. On day 16, his liver function parameters were in the cholestatic phase with decreasing transaminase levels. He tested negative for COVID-19 PCR twice, and the patient was eventually shifted to the general ward.

## Discussion

We reported a case of acute HBV reactivation in a COVID-19 patient with mental disturbances to the emergency department. The underlying pathogenesis of transient reactivation of HBV in COVID-19 remains unclear. This case illustrates the importance of an index of suspicion of acute liver injury in patients with COVID-19, as many present with varying degrees of liver test-result abnormalities [12-14]. A recent meta-analysis of 35 studies identified the pooled prevalence of abnormal liver functions in COVID-19 patients was 19% (95% confidence interval: 9-32%) [16]. Hitherto, no cases of acute hepatitis and liver failure in COVID-19 patients have been reported. Our patient tested positive for SARS-CoV-2 RNA in nasopharyngeal swab tests, even though he had no fever or respiratory symptoms. We believe that this asymptomatic patient might have been capable of transmitting the virus as he was not isolated in the emergency department. 

Our patient had an atypical presentation (altered mental status), which caused the reactivation of HBV, confirmed with strong immunological evidence. COVID-19-affected patients with digestive symptoms may require a longer time for the onset of their symptoms, delaying diagnosis considerably, as these symptoms are nonspecific [17]. Given the high burden of liver diseases globally, it is believed that underlying liver conditions such as chronic HBV infection could be reactivated, contributing to elevated liver enzyme abnormalities in COVID-19 [9, 18]. However, case studies of the interaction between pre-existing liver conditions and COVID-19 require meticulous evaluation. A recent review concluded that digestive symptoms and abnormal liver enzymes may play an important role in a large number of patients with COVID-19, particularly in those with atypical symptoms [9]. Further studies are warranted to better understand the cause of liver injuries in patients with pre-existing liver diseases who have recently contracted COVID-19.

Elevated liver-related enzymes are reported in a substantial proportion of patients with COVID-19 [2, 4, 5, 7, 10]. Our patient had elevated levels of liver aminotransferases, such as AST, ALT, and gamma-glutamyl transferase (GGT), during the subclinical phase. Contrastingly, Guan et al. identified an increased prevalence of abnormal liver aminotransferase levels, higher AST levels, and liver injury in severe cases of COVID-19 in a large cohort of 1099 patients. They observed elevated ALT (22%), AST (21%), and total bilirubin (10%) levels in patients affected with COVID-19 [1]. In the Fan et al. study, patients, specifically males, with abnormal liver test results, were more likely to have a moderate to a high degree of fever [19]. In contrast, our patient was hypothermic at admission and showed very high levels of liver enzymes, total bilirubin, and ammonia and INR value.

## Conclusions

In conclusion, HBV reactivation was induced by COVID-19 in a young patient presenting with altered mental status and elevated liver enzyme levels. This indicates the liver might be a target organ for COVID-19 and the patients with abnormal liver functions tend to have a higher risk for SARS-CoV-2. Increased attention should be paid to care these patient groups, particularly, presentation of acute hepatitis warrants isolation and testing for SARS-CoV-2 RNA in the COVID era.

## References

[1] Guan WJ, Ni ZY, Hu Y (2020). Clinical characteristics of coronavirus disease 2019 in China. N Engl J Med.

[2] Cheung KS, Hung IF, Chan PP (2020). Gastrointestinal manifestations of SARS-CoV-2 infection and virus load in fecal samples from the Hong Kong cohort and systematic review and meta-analysis (IN PRESS). Gastroenterology.

[3] Klopfenstein T, Kadiane-Oussou NJ, Royer P-Y, Toko L, Gendrin V, Zayet S (2020). Diarrhea: an underestimated symptom in coronavirus disease 2019 (IN PRESS). Clin Res Hepatol Gastroenterol.

[4] An P, Chen H, Jiang X (2020). Clinical features of 2019 novel coronavirus pneumonia presented gastrointestinal symptoms but without fever onset (PREPRINT). Lancet.

[5] Huang C, Wang Y, Li X (2020). Clinical features of patients infected with 2019 novel coronavirus in Wuhan, China. Lancet.

[6] Wang D, Hu B, Hu C (2020). Clinical characteristics of 138 hospitalized patients with 2019 novel coronavirus-infected pneumonia in Wuhan, China. JAMA.

[7] Jin X, Lian JS, Hu JH (2020). Epidemiological, clinical and virological characteristics of 74 cases of coronavirus-infected disease 2019 (COVID-19) with gastrointestinal symptoms. Gut.

[8] D’Amico F, Baumgart DC, Danese S, Peyrin-Biroulet L (2020). Diarrhea during COVID-19 infection: pathogenesis, epidemiology, prevention, and management (IN PRESS). Clin Gastroenterol Hepatol.

[9] Agarwal A, Chen A, Ravindran N, To C, Thuluvath PJ (2020). Gastrointestinal and Liver Manifestations of COVID-19. J Clin Exp Hepatol.

[10] Lin L, Jiang X, Zhang Z (2020). Gastrointestinal symptoms of 95 cases with SARS-CoV-2 infection. Gut.

[11] Pan L, Mu M, Yang P (2020). Clinical characteristics of COVID-19 patients with digestive symptoms in Hubei, China: a descriptive, cross-sectional, multicenter study. Am J Gastroenterol.

[12] Join AB, Partner AI, Edge AL (2020). COVID-19 clinical insights for our community of gastroenterologists and gastroenterology care providers. https://gi.org/2020/03/15/joint-gi-society-message-on-covid-19/.

[13] Xu XW, Wu XX, Jiang XG (2020). Clinical findings in a group of patients infected with the 2019 novel coronavirus (SARS-Cov-2) outside of Wuhan, China: retrospective case series. BMJ.

[14] Shi H, Han X, Jiang N (2020). Radiological findings from 81 patients with COVID-19 pneumonia in Wuhan, China: a descriptive study. Lancet Infect Dis.

[15] Chai X, Hu L, Zhang Y (2020). Specific ACE2 expression in cholangiocytes may cause liver damage after 2019-nCoV infection (PREPRINT). BioRxiv.

[16] Mao R, Qiu Y, He JS (2020). Manifestations and prognosis of gastrointestinal and liver involvement in patients with COVID-19: a systematic review and meta-analysis. Lancet Gastroenterol Hepatol.

[17] Chen D, Xu W, Lei Z (2020). Recurrence of positive SARS-CoV-2 RNA in COVID- 19: a case report. Int J Infect Dis.

[18] Zhang C, Shi L, Wang FS (2020). Liver injury in COVID- 19: management and challenges. Lancet Gastroenterol Hepatol.

[19] Fan Z, Chen L, Li J (2020). Clinical features of COVID-19-related liver damage. Clin Gastroenterol and Hepatol.

